# Comparison of a New In-House and Three Published HLA-B*15:*02* Screening Methods for Prevention of Carbamazepine-Induced Severe Drug Reactions

**DOI:** 10.1371/journal.pone.0155907

**Published:** 2016-05-19

**Authors:** Kanoot Jaruthamsophon, Thanya Sripo, Chonlaphat Sukasem, Pornprot Limprasert

**Affiliations:** 1 Division of Human Genetics, Department of Pathology, Faculty of Medicine, Prince of Songkla University, Hat Yai, Songkhla, Thailand; 2 Division of Pharmacogenomics and Personalized Medicine, Department of Pathology, Faculty of Medicine, Ramathibodi Hospital, Mahidol University, Bangkok, Thailand; 3 Laboratory for Pharmacogenomics, Somdech Phra Debaratana Medical Center (SDMC), Faculty of Medicine, Ramathibodi Hospital, Mahidol University, Bangkok, Thailand; Centers for Disease Control and Prevention, UNITED STATES

## Abstract

Currently, there are three published HLA-B*15:*02* screening methods for prevention of carbamazepine-induced severe drug reactions in Asian populations. To analyze available HLA-B*15:*02* screening methods, we compared four screening methods, including a multiplex PCR method, a nested PCR method, a LAMP method and our new in-house PCR-dot blot hybridization method. These methods were reviewed regarding their sensitivity, specificity, false positivity and technical considerations. Possible false positive (FP) alleles and genotypes were checked regarding the primers and probes designs, using the IMGT/HLA database. Expected FP rates in Asian populations were predicted using the Allele Frequencies Net Database. All methods had a sensitivity of more than 99.9%, although giving FP results to certain very rare alleles and genotypes. The multiplex PCR method was the only test that gave FP results to certain genotypes of HLA-B*15:*13*, the allele which is prevalent in Southeast Asian populations. In conclusion, the nested PCR, LAMP and our in-house methods could be applied in various Asian populations, but the multiplex PCR, or any test with FP to HLA-B*15:13, should be applied with caution in the Southeast Asian populations.

## Introduction

The HLA-B*15:*02* allele is known as the most significant human leukocyte antigen marker associated with carbamazepine (CBZ)-induced Stevens-Johnson syndrome and toxic epidermal necrolysis in various Asian populations [1,2]. There are a number of studies confirming the usefulness of screening for this marker before CBZ prescription, in terms of prevention of severe adverse drug reactions and cost-effectiveness [3].

Until recently, three methods were published for the identification of the HLA-B*15:*02* allele, including one multiplex PCR using 4 pairs of primers [4,5], one loop-mediated isothermal amplification (LAMP) method [6], and one nested PCR method [7]. In this study, we presented a new in-house method which we had designed, validated and implemented in our own setting, and compared the characteristics of each test to consider its applicability in medical practice.

## Materials and Methods

### In-house method

Our in-house method is based on a 2-step technique using allele-specific PCR (AS-PCR) and direct dot blot hybridization (DDB). We adopted the previously described primers, outer forward (F1) and inner reverse primer (R2) in the nested PCR method [7]. A pair of primers of the ATL1 locus in the *FMR1* gene [8] were applied as an internal control. The specific HLA-B*15:*02* and control PCR products were 137 and 301 bp, respectively. The 3’digoxigenin probe using in the DDB was designed as shown in Table 1.

**Table 1:** Sequences of primers and probe used for HLA-B*15:*02* screening by AS-PCR and DDB.

Primer	Sequence
HLA-B-F1^a^	5’- GCG AGT CCG AGG ATG GC -3’
HLA-B-R2^a^	5’- TTG TAG TAG CCG CGC AGG T -3’
ATL1-F^b^	5’- CCC TGA TGA AGA ACT TGT ATC TC -3’
ATL1-R^b^	5’- GAA ATT ACA CAC ATA GGT GGC ACT -3’
PSU HLA-B Probe	5’- GGA ACA CAC AGA TCT CCA AGA -3’- DIG^c^

^a^HLA-B-F1 and HLA-B-R2 primers provide a PCR product of 137 base pairs.

^b^ATL1-F and ATL1-R primers provide a PCR product of 301 base pairs.

^c^DIG: digoxigenin

**Fig 1:**
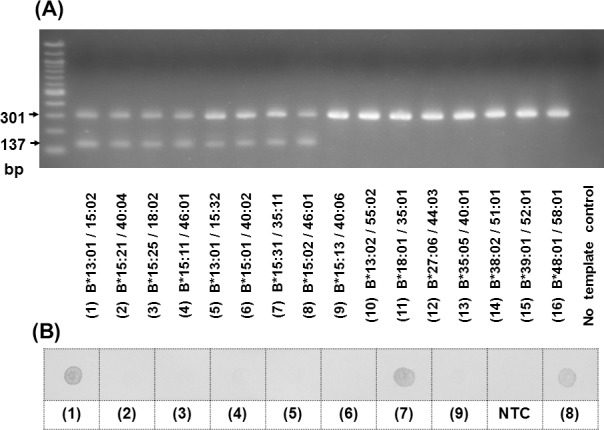
Results of AS-PCR and DDB. **(a)** Electrophoresis was applied by using 2.5% agarose gel with 100V for 30 minute. A control product was 301 bp long while the specific PCR product was 137 bp long. The alleles with PCR product included HLA-B*15:*02* (No. 1 and 8), B*15:*21* (No. 2), B*15:*25* (No. 3), B*15:*11* (No. 4), B*15:*32* (No. 5), B*15:*01* (No. 6), B*15:*31* (No. 7), and B*46:*01* (No. 4, 8). HLA-B*15:*13* (No. 9) and other common alleles (No. 10–16) gave negative results in the PCR step. **(b)** The samples analyzed in the PCR step (No. 1–9) were subsequently examined by DDB. (NTC: “no template control”).

There were two main steps in our in-house testing method, AS-PCR and DDB. For the AS-PCR step, the PCR was done in a 20 μl volume containing 100 ng of DNA template, 1X PCR buffer (Invitrogen), 2 mmol/L MgCl_2_, 0.2 mmol/L of dNTPs, 1 U of Taq polymerase (Invitrogen), 0.4 μmol/L of specific primers, and 0.2 μmol/L of control primers. The PCR step was programmed as follows: 95°C for 5 minutes, 35 cycles at 95°C, 59°C, and 72°C, each for 30 seconds. The samples with positive PCR results (Fig 1) were then tested in the second step.

In the DDB step, a testing blot was prepared by immobilizing 2.5 μl of PCR product onto a positive-charged nylon membrane. The blot was then tested with the 3’-digoxiginin labeled probe at 55°C and incubated with an alkaline phosphatase conjugated anti-digoxigenin antibody (Roche Diagnostics). Finally, the hybridization signal was developed by using chromogenic reaction.

Our method was validated by 155 anonymous leftover DNA samples which were HLA-typed by the gold standard method, sequenced-based typing. These *HLA*-*B* typing results were concealed from the investigator who performed the test. The study was carried out from October 2012 to September 2014, and was approved by the Research Ethics Committee, Faculty of Medicine Prince of Songkla University (EC no. 55-361-05-1-3). Patient informed consent was waived with the approval of the Research Ethics Committee, Faculty of Medicine Prince of Songkla University.

### Test Comparison

The three currently available HLA-B*15:*02* screening methods and our in-house methods were reviewed for their sensitivity and specificity. The false positive (FP) alleles and genotypes of each method were checked, regarding their primer sequences, using the Probe and Primer Search Tool, IMGT/HLA database [9]. Frequencies of FP results of each test were estimated from Chinese and Southeast Asian populations using the Allele Frequency Net Database [10]. We also reviewed technical considerations, required time and reagents cost of each method.

## Results

### Validation of the in-house method

**Fig 2:**
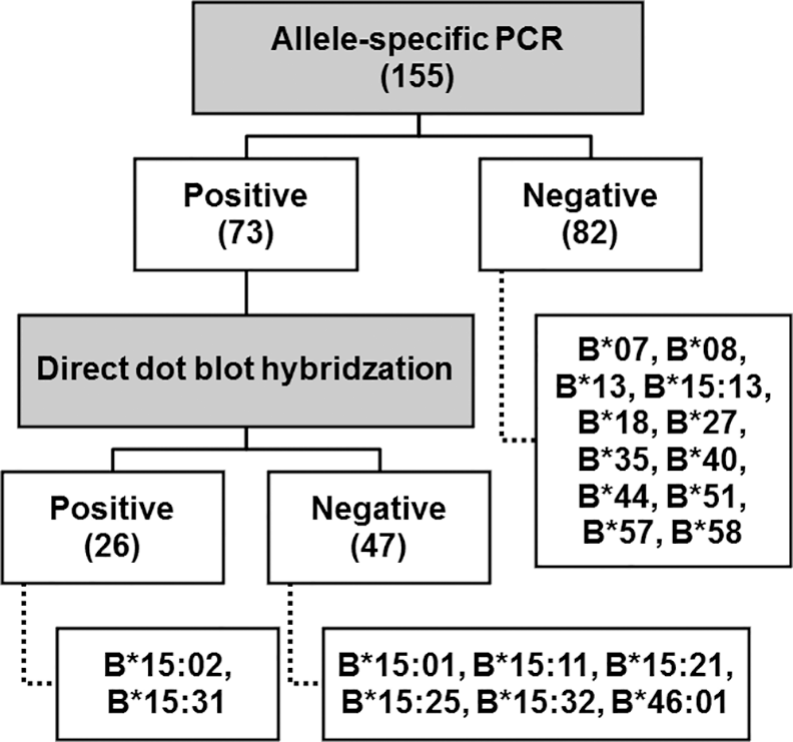
A diagram of test results and their representative alleles. A diagram demonstrated the number of samples grouped by their results and the alleles which could give each result. In the AS-PCR step, all samples were tested: 82 gave negative results and were reported as negative for screening, following which 73 positive AS-PCR samples were then tested in the DDB step, giving 47 negative and 26 positive results. The only falsely screened result was from HLA-B*15:*31*.

The AS-PCR step showed 73 positive results (2 bands of 301 bp internal control and 137 bp specific HLA-B*15:*02* PCR products); these samples were then tested by DDB. The remaining 82 samples were reported as negative (1 band of 301 bp internal control). The negative PCR results included many common alleles, i.e. *HLA*-B*07, B*13, B*27, B*40, B*44, B*58, while the negative DDB results included HLA-B*46:*01* and some HLA-B*15 alleles (Fig 2). All negative screening results were consistent with the PCR-SBT results. In the DDB step, the results identified 26 positives for HLA-B*15:*02* screening out of 73 samples. Of those 26 positive results, one sample with HLA-B*15:*31* –very rare allele in Asians [10]–was a false positive allele. Thus, the sensitivity and specificity of the new HLA-B*15:*02* screening method were 100% and 99.23%, respectively.

### Test Comparison

According to the reported validation results, the sensitivity of nested PCR, LAMP and our in-house methods were greater than 99.9% (Table 2). However, there was no validation result of the multiplex PCR method. Our analysis by the IMGT/HLA database showed that the sequences of primers and probes used in each method were matched to the HLA-B*15:*02*:*01* allele, the predominant variant of HLA-B*15:*02*. Thus, all methods gave ~100% sensitivity theoretically. For possible FP results, the set of probes and primers using in the nested PCR, LAMP and our in-house methods were not completely matched to any *HLA-B* allele or genotype with a frequency of more than 1% in Asian populations. Meanwhile, the multiplex PCR method was found to give FP results from subjects with heterozygous genotypes, in a combination of HLA-B*15:*13* with a number of HLA-B*15 alleles (Table 2). The predicted FP rates from these combinations were 4–9% in Malaysian and Indonesian populations.

**Table 2:** Comparison of test characteristics of HLA-B*15:*02* screening methods.

Method	Sample size (n), Sensitivity (Sn), Specificity (Sp)	False positive alleles and genotypes	False positive rate in Asian populations	Technical considerations	Time, reagents cost^a^
Multiplex PCR [4,5]	n: N/A, Sn: N/A, Sp: N/A	A heterozygous carrier of B*15:*13* and any of these following alleles: B*15:*01*, B*15:*11*, B*15:*12*, B*15:*21*, B*15:*25*, B*15:*27*, B*15:*32*, B*15:*35*	Depends on frequencies of B*15:*13* and other B*15 alleles	Allele dropout	~120 min, ~5.2 USD
LAMP [6]	n: 400, Sn: ~100%, Sp: >99.9%	A heterozygous carrier of B*15:*15* and B*15:*25*	Less than 1%	Possible contamination due to its highly sensitive amplification	~45 min, ~3.8 USD
Nested PCR [7]	n: 200, Sn: ~100%, Sp: >99.9%	A group of rare B*15 alleles	Less than 1%	Possible contamination during re-amplification step	1^st^ step: ~180 min, ~5.2 USD 2^nd^ step: ~60 min, ~1.3 USD
AS-PCR and DDB (this study)	n: 155, Sn: ~100%, Sp: 99.23%	B*15:*31*^*b*^ and a group of rare B*15 alleles	Less than 1%	Time consuming	1^st^ step: ~120 min, ~5.2 USD 2^nd^ step: ~180 min, ~1.8 USD

AS-PCR, allele-specific PCR; DDB, direct dot blot hybridization; N/A, not available.

Sample size (n) represents the sample size used for the validation in each test.

^a^The reagent cost of LAMP test was cited according to the previously published data [6]. The costs of other tests were calculated based on PCR-based techniques.

^b^Frequency = 0.001–0.006, according to the Allele Frequency Net Database

For technical considerations, the multiplex PCR, nested PCR and our in-house methods were relatively time-consuming compared to the LAMP method. However, the LAMP and nested PCR methods have a high risk of cross-contamination between samples due to the LAMP’s highly sensitive amplification and the nested PCR’s re-amplification steps. The cross-contamination of these amplified products may lead to FP results. However, these effects on the specificity depend on the technical skill of the operator, and thus the problem will be minimal if the test is performed by a skilled operator. Herein, we compared only the reagent costs and not the overall test costs, because the wages of laboratory personnel can vary considerably among countries.

## Discussion

Our in-house method was designed using a direct dot blot hybridization technique–even though it is relatively outdated–because this method can handle many samples at once, has fewer FPs compared to the multiplex PCR design, and has less contamination compared to the LAMP and nested PCR methods. In our practice, we found the average test time was actually even much lower than shown in Table 2, since about a half of the cases had a negative result in the first PCR step. Moreover, dot blot hybridization technique does not require an expensive equipment and it is widely used for mutation analysis of thalassemia, a common genetic disease in Asians.

As the HLA allele frequencies are different in various populations, these differences should be considered before implementing any HLA screening test in any particular setting to ensure that any FP would not appear unexpectedly. For example, the allele that gives FPs in the multiplex PCR method, HLA-B*15:*13*, is very rare in East Asia but not uncommon in Southeast Asia; its frequency can exceed 0.12 in Malaysia, Indonesia and Singapore [10]. Although any of the compared methods could be an option for HLA-B*15:*02* screening, not only the cost of the test and technical considerations, but FP results need to also be considered in terms of cost-effectiveness. Patients with these FP results may be given an alternative drug instead of CBZ. This problem could have effects on both clinical and economic aspects since CBZ could be effective and inexpensive. In Thailand, a cost-effectiveness study assigned the screening cost as 1,000 Thai Baht (~30 US Dollars) with a test specificity of 98.7%, i.e. a 1.3% FP rate [3]. If an HLA-B*15:*02* screening test which gives a FP to HLA-B*15:*13* is applied in Malaysian or Indonesian or Southern Thai (mixed Thai-Malaysian) populations then the FP rates increase, approaching 1.3% or even higher, and the cost-effectiveness is reduced. This problem would not only occur in the mentioned populations but also possibly in any populations in which the actual *HLA-B* allele frequencies have never been studied.

Since CBZ-induced SJS and TEN occur by a direct pharmacological interaction between carbamazepine and the HLA-B*15:02 molecule which activates particular types of T cell receptors [11,12], clinicains should be aware of the association between HLA-B*15:*02* and CBZ-induced SJS and TEN in any Asian population whose HLA-B*15:*02* frequency is high. In contrast, the association was not found in Japanese [13] and Korean populations [14] whose HLA-B*15:*02* frequency is very low. This is a controversial issue regarding the FDA recommendation for HLA-B*15:*02* testing before CBZ prescription in patients with ancestry across broad areas of Asia, including South Asian Indians. A suggestion has been raised the recommendation should be modified to reflect current medical evidence that not all Asians need to be screened for HLA-B*15:*02* [15]. In spite of this questionable issue and limited association studies which have not covered all Asian populations yet, we recommend that HLA-B*15:*02* screening before CBZ prescription should be implemented in all Southeast Asian populations for three reasons. Firstly, a relationship between HLA-B*15:*02* and CBZ-induced SJS and TEN has been reported from four countries in this region, Thailand, Malaysia, Singapore, and Vietnam [2,16,17]. Another report has also found a case with CBZ-induced SJS/ TEN overlap sydrome from Cambodia, in which the case was also found to have HLA-B*15:*02* [18]. Secondly, people in this region are closely related, and have been shown to share similar genetic backgrounds based on an SNP study [19]. And finally, Southeast Asians also share high frequencies of HLA-B*15:*02*.

## Conclusion

All screening methods had a high sensitivity (> 99%), however, only three methods (the nested PCR, LAMP and our in-house methods) gave no FPs in identifying HLA-B*15:*13*. These three methods, therefore, can be applied widely in different ethnic Asian populations. Ultimately, health care providers need to decide which screening method is appropriate for their own settings.
